# Supplementary material for: The “ready-to-hand” test: Diagnostic availability and usability in primary health care settings in Sierra Leone
Source: PLOS Glob Public Health. 2023 Feb 10;3(2):e0000604. doi: 10.1371/journal.pgph.0000604 (PMC10021322; doi:10.1371/journal.pgph.0000604)
Supplement: S1 Data — (DOCX) [file pgph.0000604.s001.docx]

**S1 Data**

**Table A: Availability of RDTs**

| Tests | Total availability *n*/N (%) | Rural availability *n*/N (%) | Urban availability *n*/N (%) | Government supply n/N (%) | Government/private supply n/N (%) | Private supply n/N (%) | *Urban-rural comparison (p*-value/φ*_c_*) | Lab technician comparison (*p*-value/φ*_c_*) |
| --- | --- | --- | --- | --- | --- | --- | --- | --- |
| Malaria RDT** (Global fund) | 39/40 (97.5%) | 14/15 (93.3%) | 25/25 (100.0%) | 36/39 (92.3%) | 1/39 (2.6%) | 2/39 (5.1%) | FET = 0.375  **φ*_c_*** = 0.207 | FET = 1.000  **φ*_c_*** = 0.086 |
| Combined HIV/Syphilis RDT**  (Global fund) | 31/40 (77.5%) | 10/15 (66.7%) | 21/25 (84.0%) | 27/30* (90.0%) | 0/30* (0.0%) | 3/30* (10.0%) | FET = 0.255  **φ*_c_*** = 0.283 | FET = 0.168  **φ*_c_*** = 0.283 |
| HIV I/II RDT** (Global fund) | 27/40 (67.5%) | 10/15 (66.7%) | 17/25 (68.0%) | 24/27 (88.9%) | 0/27 (0.0%) | 3/27 (11.1%) | FET = 1.000  **φ*_c_*** = 0.014 | FET = 1.000  **φ*_c_*** = 0.014 |
| Urine pregnancy test RDT** | 23/40 (57.5%) | 10/15 (66.7%) | 13/25 (52.0%) | 8/23 (34.8%) | 2/23 (8.7%) | 13/23 (56.5%) | χ² = 0.364  **φ*_c_ =*** 0.144 | FET = 0.256  **φ*_c_*** = 0.221 |
| Random blood sugar RDT** | 21/40 (52.5%) | 9/15 (60.0%) | 12/25 (48.0%) | 3/21 (14.3%) | 1/21 (4.7%) | 17/21 (81.0%) | χ² = 0.462  **φ*_c_*** = 0.116 | FET = 0.021  **φ*_c_*** = 0.393 |
| Urine glucose RDT** | 17/40 (42.5%) | 8/15 (53.3%) | 9/25 (36.0%) | 5/17 (29.4%) | 1/17 (5.9%) | 11/17 (64.7%) | χ² = 0.283  **φ*_c_ =*** 0.170 | FET = 0.134  **φ*_c_*** = 0.263 |
| Haemoglobin RDT** | 15/40 (37.5%) | 8/15 (53.5%) | 7/25 (28.0%) | 4/15 (26.7%) | 0/15 (0.0%) | 11/15 (73.3%) | χ² = 0.109  **φ*_c_*** = 0.253 | FET = 0.008  **φ*_c_*** = 0.448 |
| Urine analysis RDT** | 15/40 (37.5%) | 7/15 (46.7%) | 8/25 (32.0%) | 1/14* (7.1%) | 2/14* (14.3%) | 11/14*(78.6%) | χ² = 0.354  **φ*_c_*** = 0.147 | FET = 0.008  **φ*_c_*** = 0.448 |
| Cholera RDT | 13/40 (32.5%) | 3/15 (20.0%) | 10/25 (40.0%) | 13/13 (100.0%) | 0/13 (0.0%) | 0/13 (0.0%) | FET = 0.298  **φ*_c_*** = 0.207 | FET = 1.000  **φ*_c_*** = 0.010 |
| Hepatitis B RDT** | 10/40 (25.0%) | 7/15 (46.7%) | 3/25 (12.0%) | 2/10 (20.0%) | 0/10 (0.0%) | 8/10 (80.0% | FET = 0.024  **φ*_c_*** = 0.388 | FET = 0.029  **φ*_c_*** = 0.380 |
| Hepatitis B profile RDT | 7/40 (17.5%) | 3/15 (20.0%) | 4/25 (16.0%) | 0/7 (0.0%) | 0/7 (0.0%) | 7/7 (100.0%) | FET = 1.000  **φ*_c_*** = 0.051 | FET = 0.034  **φ*_c_*** = 0.382 |
| Hepatitis C RDT** | 6/40 (15.0%) | 2/15 (13.3%) | 4/25 (16.0%) | 2/6 (33.3%) | 0/6 (0.0%) | 4/6 (66.7%) | FET = 1.000  **φ*_c_*** = 0.036 | FET = 1.000  **φ*_c_*** = 0.059 |
| Syphilis RDT** | 4/36* (11.1%) | 3/14* (21.4%) | 1/22* (4.5%) | 1/4 (25.0%) | 0/4 (0.0%) | 3/4 (75.0%) | FET = 0.277  **φ*_c_*** = 0.262 | FET = 0.255  **φ*_c_*** = 0.204 |
| Helicobacter pylori RDT | 4/40 (10.0%) | 2/15 (13.3%) | 2/25 (8.0%) | 1/4 (25.0%) | 0/4 (0.0%) | 3/4 (75.0%) | FET = 0.622  **φ*_c_*** = 0.086 | FET = 0.030  **φ*_c_*** = 0.419 |
| Faecal occult blood RDT | 2/40 (5.0%) | 2/15 (13.3%) | 0/25 (0.0%) | 0/2 (0.0%) | 0/2 (0.0%) | 2/2 (100.0%) | FET = 0.135  **φ*_c_*** = 0.296 | FET = 0.404  **φ*_c_*** = 0.151 |
| Leishmaniasis RDT** | 0/40 (0.0%) | 0/15 (0.0%) | 0/25 (0.0%) | 0/0 (0.0%) | 0/0 (0.0%) | 0/0 (0.0%) | NA | NA |
| Haemoglobin glycated (HbA1c) RDT** | 0/40 (0.0%) | 0/15 (0.0%) | 0/25 (0.0%) | 0/0 (0.0%) | 0/0 (0.0%) | 0/0 (0.0%) | NA | NA |

Data is presented in absolute number of CHCs (percentage of sample). FET = Fisher’s Exact Test, χ² = Chi square test, **φ*_c_*** = Cramér’s V. * Missing data. **These tests are listed in the 2019 EDL or 2021 EDL. NA = No test availability.

**Table B: Reported availability of manual assays**

| Tests | Total availability *n*/N (%) | Rural availability *n*/N (%) | Urban availability *n*/N (%) | Government supply n/N (%) | Government/private supply n/N (%) | Private supply n/N (%) | *Urban-rural comparison (p-*value/φ*_c_*) | Lab technician comparison (*p*-value/φ*_c_*) |
| --- | --- | --- | --- | --- | --- | --- | --- | --- |
| Widal test (typhoid) | 15/40 (37.5%) | 8/15 (53.3%) | 7/25 (28.0%) | 0/15 (0.0%) | 0/15 (0.0%) | 15/15 (100.0%) | χ² = 0.109  **φ*_c_*** = 0.253 | FET = 0.001  **φ*_c_*** = 0.572 |
| Biological specimen routine analysis (stool/urine) | 14/40 (35.0%) | 8/15 (53.3%) | 6/25 (24.0%) | 2/13* (15.4%) | 5/13* (38.5%) | 6/13*(46.1%) | χ² = 0.060  **φ*_c_*** = 0.298 | FET = 0.044  **φ*_c_*** = 0.358 |
| TB sputum microscopy *** | 14/40 (35.0%) | 8/15 (53.3%) | 6/25 (24.0%) | 14/14 (100.0%) | 0/14 (0.0%) | 0/14 (0.0%) | χ² = 0.060  **φ*_c_*** = 0.298 | FET = 0.044  **φ*_c_*** = 0.358 |
| Sickle cell disease** | 12/40 (30.0%) | 7/15 (46.7%) | 5/25 (20.0%) | 1/12 (8.3%) | 2/12 (16.7%) | 9/12 (75.0%) | FET = 0.091  **φ*_c_*** = 0.282 | FET = 0.097  **φ*_c_*** = 0.300 |
| Malaria microscopy*** | 11/40 (27.5%) | 7/15 (46.7%) | 4/25 (16.0%) | 3/11 (27.3%) | 1/11 (9.1%) | 7/11 (63.6%) | FET = 0.065  **φ*_c_*** = 0.332 | FET = 0.007  **φ*_c_*** = 0.473 |
| Skin snip | 10/40 (25.0%) | 6/15 (40.0%) | 4/25 (16.0%) | 1/10 (10.0%) | 1/10 (10.0%) | 8/10 (80.0%) | FET = 0.135  **φ*_c_*** = 0.268 | FET = 0.003  **φ*_c_*** = 0.518 |
| Blood typing** | 9/40 (22.5%) | 4/15 (26.7%) | 5/25 (20.0%) | 0/9 (0.0%) | 0/9 (0.0%) | 9/9 (100.0%) | FET = 0.705  **φ*_c_*** = 0.077 | FET = 0.001  **φ*_c_*** = 0.713 |
| Blood microscopy | 2/40 (5.0%) | 2/15 (13.3%) | 0/25 (0.0%) | 2/2 (100.0%) | 0/2 (0.0%) | 0/2 (0.0%) | FET = 0.135  **φ*_c_*** = 0.296 | FET = 1.000  **φ*_c_*** = 0.124 |
| Fungal (yeast/mould) identification | 2/40 (5.0%) | 0/15 (0.0%) | 2/25 (8.0%) | 2/2 (100.0%) | 0/2 (0.0%) | 0/2 (0.0%) | FET = 0.519  **φ*_c_*** = 0.178 | FET = 0.404  **φ*_c_*** = 0.151 |
| White blood cell count*** | 1/40 (2.5%) | 1/15 (6.7%) | 0/25 (0.0%) | 0/1 (0.0%) | 0/1 (0.0%) | 1/1 (100.0%) | FET = 0.375  **φ*_c_*** = 0.207 | FET = 0.225  **φ*_c_*** = 0.297 |
| CD4 count** | 0/40 (0.0%) | 0/15 (0.0%) | 0/25 (0.0%) | 0/0 (0.0%) | 0/0 (0.0%) | 0/0 (0.0%) | NA | NA |
| Prostate monitoring | 0/40 (0.0%) | 0/15 (0.0%) | 0/25 (0.0%) | 0/0 (0.0%) | 0/0 (0.0%) | 0/0 (0.0%) | NA | NA |

Data is presented in absolute number of CHCs (percentage of sample). FET = Fisher’s Exact Test, χ² = Chi square test, φc = Cramér’s V. *Missing data. **These tests are listed in the 2019 EDL or 2021 EDL. *** These tests were listed in 2018 EDL version for places without a laboratory, but no longer in the 2019/2021 EDL for places without a laboratory. NA = No test availability.

**Fig A: Availability of equipment, consumables and reagents**

**Fig B: Availability of laboratory infrastructure**
