# Supplementary material for: The “ready-to-hand” test: Diagnostic availability and usability in primary health care settings in Sierra Leone
Source: PLOS Glob Public Health. 2023 Feb 10;3(2):e0000604. doi: 10.1371/journal.pgph.0000604 (PMC10021322; doi:10.1371/journal.pgph.0000604)
Supplement: S1 Text — (DOCX) [file pgph.0000604.s002.docx]

**S1 Text**

**Checklist for assessing diagnostics available at Community Health Centres**

1. **.   Basic information**

Notes

| Name of the Community Health Centre |  |
| --- | --- |
| Address |  |
| District |  |
| City/Town/Village/Chiefdom |  |
| CHC team lead |  |

1. .   Hours of service provided by Community Health Centre?

- 24x7
- Others (specify)

1. . a. Number of CHP & MCHP covered by the Community Health Centre:

b. Mobility of CHC to cover CHP&MCHP is by ?

☐Motorbike

☐Bicycle

☐Government Vehicle

☐Public Vehicles

☐Others………………….

1. . Location of the Community Health Centre:
2. . Approximate population covered by the Community Health Centre:
3. . Which district hospital is the nearest to the CHC: Name:

Approx distance: kms

1. . Does the CHC have a laboratory?

- Yes
- No

7a. If yes, timings of laboratory: Open from …………. until ……………. hours

7b. If no, are diagnostic tests outsourced to a specific private laboratory? (If yes, specify):

…………………………………………………………………………………………….

7c. If no, are specimens sent to another public facility for testing? (If yes, please specify)

……………………………………………………………………………………………

7d. If yes, are all tests done in the same place, if not where else in the CHC are tests done?

.........................................................................................................................................................

**8. Facility description**

8a. Describe the exterior of the facility (size, design, paintwork, signage, any porch/ veranda)

8b. What materials is the facility building made from and what condition are they in?

8c. Where is the facility located (e.g. on main road/ side street/ next to church, mosque / on residential street etc.) and what other buildings or facilities are nearby?

8d. Describe the inside of the facility (layout, number and size of rooms, furnishings, windows, heat, smells, lighting)

8e. Any relevant background/context to facility (e.g. When it was opened, what services it provides (maternity, delivery, feeding programmes, NGO?), was the facility functioning during Ebola times? Any changes in services during Ebola times? Recent closures or changes in staffing, any recent events for national/state programmes. Anything else that marks it out as distinct from other facilities.)

# 9. Availability and Quality: Tests available at this moment, at the Community Health Centre laboratory

# Purpose of the checklist is to observe and verify^[[1]](#endnote-1)^ if the tests is available; it requires the lab technician to show the test to the observer.

# Please ask to make a picture of each test, consumable, reagent, so that you can note down the name of manufacturing company.

# Tests listed below are those that are listed in the Sierra Leone Basic Package of Essential Health Services 2015-2020 to be available at CHC level^[[2]](#endnote-2)^.

| **Name of the test** | **Yes/No** | **Not verified** | **If yes, method used (rapid test, specify strip or cassette)** | **If no, when last used** | **Government^[[3]](#endnote-3)^ supplied + name of manufacturing company** | **Private supplied + name of manufacturing company + supplier** | **WHO pre-qualified (Yes/No)** | **Notes** |
| --- | --- | --- | --- | --- | --- | --- | --- | --- |
| Pregnancy test RDT |  |  |  |  |  |  |  |  |
| ***Haematology*** |  |  |  |  |  |  |  |  |
| Haematology (Hb) |  |  |  |  |  |  |  |  |
| White Blood Cell Count (WBC) |  |  |  |  |  |  |  |  |
| Malaria Parasite (MP) (blood film) |  |  |  |  |  |  |  |  |
| Malaria RDT |  |  |  |  |  |  |  |  |
| Sickle cell disease (sickling) |  |  |  |  |  |  |  |  |
| Blood Grouping |  |  |  |  |  |  |  |  |
| ***Blood glucose Level (Diabetes Monitoring)*** |  |  |  |  |  |  |  |  |
| Random Blood Sugar |  |  |  |  |  |  |  |  |
| Urine glucose level |  |  |  |  |  |  |  |  |
| Haemoglobin Glycated |  |  |  |  |  |  |  |  |
| **Name of the test** | **Yes/No** | **Not verified** | **If yes, method used (rapid test, specify strip or cassette)** | **If no, when last used** | **Government supplied + name of manufacturing company** | **Private supplied + name of manufacturing company + supplier** | **WHO pre-qualified (Yes/No)** | **Notes** |
| ***Kidney Function Test***  *(appeared to be mistakenly included in Basic Package at CHC level – not supposed to be available in CHC)* | | | | | | | | |
| Urea kit (appeared to be mistakenly included in CHC |  |  |  |  |  |  |  |  |
| Uric Acid kit |  |  |  |  |  |  |  |  |
| Creatinine kit |  |  |  |  |  |  |  |  |
| Sodium kit (electrolyte) |  |  |  |  |  |  |  |  |
| ***Microbiology*** |  |  |  |  |  |  |  |  |
| Biological specimen routine analysis (specify specimen type; urine, stool) |  |  |  |  |  |  |  |  |
| Multi stick for urinalysis (dip-strip)_ |  |  |  |  |  |  |  |  |
| TB Sputum Routine for AAFB |  |  |  |  |  |  |  |  |
| Skin snip ( Oncho) |  |  |  |  |  |  |  |  |
| Leischmania diagnosis |  |  |  |  |  |  |  |  |
| Blood microscopy (Tryp, Filaria) |  |  |  |  |  |  |  |  |
| Yeast and mould identification |  |  |  |  |  |  |  |  |
| Faecal Occult Blood |  |  |  |  |  |  |  |  |
| **Name of the test** | **Yes/No** | **Not verified** | **If yes, method used (rapid test, specify strip or cassette)** | **If no, when last used** | **Government supplied + name of manufacturing company** | **Private supplied + name of manufacturing company + supplier** | **WHO pre-qualified (Yes/No)** | **Notes** |
| H Pyloric Ab Dip strip |  |  |  |  |  |  |  |  |
| HBsAg Combo (Hep b) IgG/IgM |  |  |  |  |  |  |  |  |
| HBV (hep B) profile |  |  |  |  |  |  |  |  |
| HCV (hep C)  HCV Ag-Ab) |  |  |  |  |  |  |  |  |
| Syphilis /TPHA/RPR |  |  |  |  |  |  |  |  |
| HIV rapid test |  |  |  |  |  |  |  |  |
| HIV I and II (Recombigen) |  |  |  |  |  |  |  |  |
| CD4 Count |  |  |  |  |  |  |  |  |
| Prostrate Monitoring PSA |  |  |  |  |  |  |  |  |
| ***Other not listed in basic package of essential health services*** |  |  |  |  |  |  |  |  |
| Cholera (during an outbreak) RDT & Culture |  |  |  |  |  |  |  |  |
| Widal test (semi-quantitative titre) |  |  |  |  |  |  |  |  |
| Stool microscopy (for Ova, Cysts, Protozea) |  |  |  |  |  |  |  |  |
| **Name of the test**  (any other tests not mentioned above..) | **Yes/No** | **Not verified** | **If yes, method used (rapid test, specify strip or cassette)** | **If no, when last used** | **Government supplied + name of manufacturing company** | **Private supplied + name of manufacturing company + supplier** | **WHO pre-qualified (Yes/No)** | **Notes** |
|  |  |  |  |  |  |  |  |  |
|  |  |  |  |  |  |  |  |  |
|  |  |  |  |  |  |  |  |  |
|  |  |  |  |  |  |  |  |  |
|  |  |  |  |  |  |  |  |  |
|  |  |  |  |  |  |  |  |  |
|  |  |  |  |  |  |  |  |  |
|  |  |  |  |  |  |  |  |  |
|  |  |  |  |  |  |  |  |  |

| **Reagents** | **Yes/No** | **If no, when last used** | **Government supplied + name of manufacturing company** | **Private supplied + name of manufacturing company + supplier** | **Notes** |
| --- | --- | --- | --- | --- | --- |
| Field stain A&B |  |  |  |  |  |
| Normal saline |  |  |  |  |  |
| Gram Staining Kit |  |  |  |  |  |
| Sodium Metabisulphite |  |  |  |  |  |
| Turk’s Solution (WBC) |  |  |  |  |  |
| Giemsa Stain Solution |  |  |  |  |  |
| Retic View Stain Kit |  |  |  |  |  |
| Methylene Blue |  |  |  |  |  |
| TB Ziehl-Neelsen Kit |  |  |  |  |  |
| Harris Hematoxylin and Eosin |  |  |  |  |  |
| Xylene |  |  |  |  |  |
| HCL |  |  |  |  |  |
| Absolute Ethanol |  |  |  |  | Not mentioned in basic package |
| Distilled water |  |  |  |  | Supplied by Malaria program – not in basic package |
| Grouping sera |  |  |  |  | Not mentioned in basic package |
|  |  |  |  |  |  |
|  |  |  |  |  |  |

| **Name of the Consumables** | **Yes/No** | **If no, when last used** | **Government supplied + name of manufacturing company** | **Private supplied + name of manufacturing company + supplier** | **Notes** |
| --- | --- | --- | --- | --- | --- |
| Pasteur pipette |  |  |  |  |  |
| Microscopy slides |  |  |  |  |  |
| Coverslips |  |  |  |  |  |
| Cryovial tubes |  |  |  |  |  |
| Blood collection tubes |  |  |  |  |  |
| Filtered pipettes tips |  |  |  |  |  |
| Hand Sanitizer |  |  |  |  |  |
| Hair dryer |  |  |  |  |  |
| Face shields |  |  |  |  |  |
| Plastic cuvettes |  |  |  |  |  |
| Glass cuvettes |  |  |  |  |  |
| Blood Lancet |  |  |  |  |  |
| Cotton wool |  |  |  |  |  |
| Needles and syringes |  |  |  |  |  |
| Nitrile disposable gloves |  |  |  |  |  |
| powdered-disposable gloves |  |  |  |  |  |
| Cryovial storage boxes |  |  |  |  |  |
| Beakers (50ml,250ml,500ml,1000ml) |  |  |  |  |  |
| Measuring cylinders |  |  |  |  |  |
| Test tubes |  |  |  |  |  |

| **Name of the Consumables** | **Yes/No** | **If no, when last used** | **Government supplied + name of manufacturing company** | **Private supplied + name of manufacturing company + supplier** | **Notes** |
| --- | --- | --- | --- | --- | --- |
| Graduated bottles with screw cap |  |  |  |  |  |
| laboratory tissues (absorbent) |  |  |  |  |  |
| Biohazard/disposal bags - black |  |  |  |  |  |
| Biohazard/disposal bags - yellow |  |  |  |  |  |
| Biohazard/disposal bags - red |  |  |  |  |  |
| Cool/Shipping boxes |  |  |  |  |  |
| Tourniquet |  |  |  |  |  |
| Haemocue cuvettes |  |  |  |  |  |
| Urine/stool (universal) containers |  |  |  |  |  |
| Disposable scalpel set |  |  |  |  |  |
| Micropipette sets |  |  |  |  |  |
| pH tester |  |  |  |  |  |
| WHO Haemoglobin colour match |  |  |  |  |  |
|  |  |  |  |  |  |
|  |  |  |  |  |  |

**Notes/ Comments/ Observations on diagnostics, reagents, consumables and accessories in laboratory. (e.g. any additional information on internal or external quality assurance, where was survey information sourced, where SOPs were found etc):**

**Notes: E.G: Source of survey information, were SOPs found on site**

**Comments:**

**Observations:**

**10. Equipment**

| **Equipment** | **Yes/No** | **If Yes, quantity** | **Energy source (if relevant e.g. battery; mains; generator; solar; gas)** | **Equipment in working condition (Yes or No)** |
| --- | --- | --- | --- | --- |
| Multifocal light microscope |  |  |  |  |
| Water distillation unit / deioniser |  |  |  |  |
| Refrigerator |  |  |  |  |
| Hand centrifuge |  |  |  |  |
| Centrifuge |  |  |  |  |
| Incubator |  |  |  |  |
| Autoclave |  |  |  |  |
| Sterilizer |  |  |  |  |
| Haemocue machines |  |  |  |  |
| Water bath |  |  |  |  |
| Thermometer |  |  |  |  |
| Balance |  |  |  |  |
| Gas cylinder |  |  |  |  |
| Bunsen Burner |  |  |  |  |
| Spirit lamp |  |  |  |  |
| Pipette stands |  |  |  |  |
| Manual pipette pump |  |  |  |  |
| Test tube washer |  |  |  |  |
| protective eye goggles |  |  |  |  |
| Laboratory coats |  |  |  |  |
| Sharps container |  |  |  |  |
| Dustbin |  |  |  |  |
| Fire extinguisher |  |  |  |  |
| Fire blankets |  |  |  |  |

| **Equipment continued** | **Yes/No** | **If Yes, quantity** | **Energy source (if relevant e.g. battery; mains; generator; solar; gas)** | **Equipment in working condition (Yes or No)** |
| --- | --- | --- | --- | --- |
| First aid kit |  |  |  |  |
| laboratory boots |  |  |  |  |
| laboratory head gear |  |  |  |  |
| laboratory nose mask |  |  |  |  |
| Tube racks |  |  |  |  |
| Stopwatch |  |  |  |  |
| Aprons |  |  |  |  |
| Slide racks  tibe |  |  |  |  |
| Glass dryer |  |  |  |  |
| Staining bath |  |  |  |  |
|  |  |  |  |  |
|  |  |  |  |  |
|  |  |  |  |  |
|  |  |  |  |  |

**Infrastructure^[[4]](#endnote-4)^**

1. **Maintenance of equipment in the laboratory**

**Notes**

i^[[5]](#footnote-1)^. Is there a biomedical engineer that service your laboratory equipment?

☐ Yes ☐ No

ii.Do you service your laboratory equipment?

☐ Yes ☐ No

iii.If yes, how often?

☐ Once every one year ☐ Every two years ☐ Never been serviced

☐ Any time a biomedical engineer is available

iv. if yes, list the type of equipment that gets serviced……………………………………………………………………………………………

1. Is there a maintenance schedule for the equipment, other than daily cleaning?

☐Yes ☐ No ☐ Do not know

1. Is there a maintenance record? ☐ Yes ☐ No
2. In case of a breakdown, how are repairs handled?
   - Send for repair
   - Dedicated service engineer will come onsite
   - Repair will be done locally by non-specialized engineer
   - other: A written complaint is sent to the district health management team and then it is worked upon
3. Are the records of refrigerator/freezer temperatures maintained? ☐ Yes ☐No
4. Is there a service and maintenance contract for major equipment? Yes/No

Details: ………………………………………………………………………………..

1. Do you know if each piece of (or all) equipment has a service agreement with a maintenance/service agent?

………………………………………………………………………………………………

1. how long on average does it take before repair is handled? …………………………………
2. Are used and non-functional equipment removed from the lab, or sent to a central place for destruction, or repair?.............................................................................................................…..
3. **Coverage: Test utilization at the CHC**

**Notes e.g. Where survey information sourced (e.g. availability of ledgers)/ any missing data/ anomalies.**

| **Tuberculosis** | | |
| --- | --- | --- |
| **Name of the test** | **Number of tests done in last 3 months** | **Number of suspected TB patients or contacts of confirmed TB patients visited the facility in last 3 months** |
| Sputum AFB |  |  |
| …. |  |  |
| **HIV** | | |
| **Name of the test** | **Number of tests done in last 3 months** | **Number of suspected HIV patients or contacts of confirmed HIV patients visited the facility in last 3 months** |
| HIV rapid test |  |  |
| … |  |  |
| **Malaria** | | |
| **Name of the test** | **Number of tests done in last 3 months** | **Number of patients with acute febrile illness visited the facility in last 3 months** |
| Malaria microscopy |  |  |
| Malaria RDT |  |  |
| **Hepatitis B** | | |
| **Name of the test** | **Number of tests done in last 3 months** | **Number of suspected Hep B patients visited the facility in last 3 months** |
| Hepatitis B antigen |  |  |
| **Typhoid** | | |
| **Name of the test** | **Number of tests done in last 3 months** | **Number of suspected typhoid cases visited the facility in last 3 months** |
| Widal test |  |  |
|  | | |

**Notes e.g. Where survey information sourced/ any missing data/ anomalies.**

**Any other diagnostic tests available:**

| **Maternal, Newborn and Child Health** | | |
| --- | --- | --- |
| **Name of the test** | **Number of tests done in last 3 months** | **Number of pregnant women visited the facility in last 3 months** |
| Hemoglobin estimation |  |  |
| Blood sugar |  |  |
| Urine pregnancy test |  |  |
| HIV rapid test |  |  |
| Syphilis rapid test |  |  |
| Hepatitis B rapid test (HBsAg) |  |  |
| TB symptom screening  (cough, fever, weight loss, hemopty-­‐‑ sis) |  |  |
| Tuberculin skin test* (not done at CHC according to test menu) |  |  |
| Malaria RDT |  |  |
| Sickling |  |  |
| **Type 2 diabetes** | | |
| **Name of the test** | **Number of tests done in last 3 months** | **Number of patients with suspected or high risk of type 2 diabetes visited the facility in last 3 months** |
| Blood glucose |  |  |
| Urine glucose (dipstick) |  |  |

1. Are the standard operating procedures (SOPs) available in this laboratory/maternity wing to perform the tests?

☐Yes ☐ No

1. How is the reporting of test results done in the Community Health Centre?
   1. Telephonically
   2. Through online portal
   3. Same day to the patient
   4. When patient comes back later (how many days later: )

**Notes/ Comments/ Observations (e.g. where was survey information sourced, where SOPs were found etc):**

# Human Resource available at the Community Health Centre:

**Notes e.g. Any recent changes to staffing:**

| **Staff designation** (see basic package for staffing norms) | **Available at the CHC, yes/no** | **Number of post (formal/on paper)** | **Number in post actually filled (pincode (P); on volunteer V)** |
| --- | --- | --- | --- |
| Community Health Officer (CHO) |  |  |  |
| Community Health Assistant |  |  |  |
| Public Health Aide |  |  |  |
| Environmental Health Officer |  |  |  |
| Midwife |  |  |  |
| State Enrolled Community Health Nurse (SECHN) |  |  |  |
| Maternal and Child Health Aid (MCH Aides) |  |  |  |
| Community Mental Health Aide |  |  |  |
| Laboratory technician |  |  |  |
| Laboratory assistant |  |  |  |
| Pharmacy technician |  |  |  |
| Assistant nutritionist |  |  |  |
| Porter/Cleaner |  |  |  |
| Security |  |  |  |
| Other: |  |  |  |

1. **Transportation of specimens**

**Notes e.g. Where survey information sourced/ any missing data/ anomalies.**

**What package material is used to transport specimens?**

- 1. When are the specimens transported for the tests?
  - Within 2-4 hrs of sample collection
  - Next day of sample collection
  - Within 7 days of sample collection
  1. How are the specimens transported to the other lab?
- Through courier
- Through lab attendant or any other designated person
- Through patient/patient’s attendant
- Other method (specify):
  1. When are the reports received from the other laboratory/Community Health Centre?
  - Same day
  - Next day
  - One week
  - More than week (specify):
  1. How are the reports received from the other laboratory/Community Health Centre?
  - Telephonically
  - Through email
  - Through lab attendant or any other designated person

☐ Other method (specify):

- 1. If there is a specimen transported which mode of transport is used:

**Notes**

1. By Government transport
2. By bicycle
3. By public transport
4. By Keke (Tri cycle)
5. Others………………………………………..

f. Are specimen transported in a cold chain?

☐ Yes

☐ No

1. **Electricity**
   1. Is there electricity in all parts of the Community Health Centre? ☐ In all parts

☐In some parts

☐None

- 1. Regular Power Supply ☐ Continuous Power Supply
     - Occasional power failure
     - Power cuts in summer only
     - Regular power cuts
     - No power supply
  2. Stand by facility (generator/UPS) available in working condition today

(e.g. is there fuel)? ☐Yes ☐ No

- 1. Are there renewable energy technologies at the facility (e.g. solar) ☐ Yes ☐ No
  2. Please specify____________________________________________

**Notes**

- 1. Is air-conditioner (AC) available at the laboratory? ☐Yes ☐ No
  2. Is the AC supported by stand by electricity facility? ☐Yes ☐ No
  3. Who provides electricity? (circle all relevant answers)

1. MOHS
2. WHO
3. Global Fund
4. CDC
5. CHC fund
6. Other:…………………..

i. How long do you use the fuel provided?

1. 24 hours
2. 12 hours
3. 9am to 1pm
4. 3 Hours
5. When there are samples in the laboratory

j.What source is the electricity provided?

1. Generator
2. Power plant
3. National grid
4. Solar
5. Others:……………….
6. **Water**

Any **observable** anomalies in the segregation of waste? E.g. waste in the wrong coloured bin.

Describe the condition of the burning pit/incinerator (location, is it fenced, brick enclosure on the ground, waste residues/ashes buried, any leftover glassware or other things that didn’t burn, animals around?)

Describe the condition of the waste bins in wards/lab, are they overflowing, is there waste next to the bin?

- 1. Is there a water supply at the facility? ☐Yes ☐ No
  2. Is there a sink in the laboratory? ☐Yes ☐ No
  3. Are there water purification chemicals or filter? ☐Yes ☐ No
  4. Does the facility have a running tap? ☐Yes ☐ No
  5. Does the facility have a bore hole water? ☐Yes ☐ No
  6. Does the facility have a storage tank? ☐Yes ☐ No

1. **Diagnostic waste management in the CHC laboratory**

| **Availability of waste management/disposal unit** | **Yes/No** | **Notes** |
| --- | --- | --- |
| Waste management SOP |  |  |
| Incinerator |  |  |
| burial pit |  |  |
| Septic tanks |  |  |
| Drainage system |  |  |
| Sharps containers in laboratory |  |  |
| Sharps containers in wards/consultation |  |  |
| Placenta pit |  |  |
| Sanitation facilities for patients |  |  |
| Sanitation facilities for staff |  |  |

19a. If an incinerator is present, which materials do you incinerate/autoclave……………………………………………………………………………………………………………………………...

……………………………………………………………………………………………………………………………………………………

19b. If no, how do you disinfect your pathogenic wastes? ☐ By burning ☐ In a Pit ☐ In an open dumping ground

19c. Does the community contribute in the Laboratory waste management? ☐ Yes ☐ No

19d. If yes, how does the community contribute to Laboratory waste management

……………………………………………………………………………………………………………………………………………….….

**20a. Condition of waste receptacles**

**To observe:** Segregation of waste at point of collection

a1. are there separate bins for medical waste and general waste in laboratory? ☐ Yes ☐ No

A11. Have bags been placed at the inner side of the waste bins? ☐ Yes ☐ No

A2. Is waste bin with red bag (highly infectious waste) available in the lab? ☐ Yes ☐ No

A3.Is waste bin with yellow bag (infectious waste) available in the lab? ☐ Yes ☐ No

a4. Is waste bin with black bag (general waste) available in the lab? ☐ Yes ☐ No

a5. Segregation of waste at point of final disposal: is medical waste disposed separately from general waste? ☐ Yes ☐ No

a6. Is the biohazard symbols imprinted over the waste bins? ☐ Yes ☐ No

a7. Are posters to guide users displayed near waste bins? ☐ Yes ☐ No

A8. Do laboratory staff double waste bags in a bin? ☐ Yes ☐ No

**b To observe:** mutilation of recyclable waste:

b1. Are used hypodermic needles destroyed? ☐ Yes ☐ No

b2. Is nozzle of syringes destroyed? ☐ Yes ☐ No

b3. Are used hypodermic needles found re-capped? ☐ Yes ☐ No

b4. Are used hypodermic needles found bent? ☐ Yes ☐ No

B5. Are sharp bins provided at this facility? ☐ Yes ☐ No

B6 How are dipsticks disposed? (circle which is relevant based on observation in laboratory)

1. Dipstick place in chlorine solution and placed in a yellow disposable bag
2. Dipstick placed in a black disposable bag
3. Dipstick placed in the bin
4. Dipstick placed in a plain plastic bag

**Questions to ask:**

a8. Who is responsible for disposing of waste? …………………………………….

a9. How often is waste disposed in incinerator/pit? ☐ Everyday ☐ weekly ☐ monthly

a10. Who provides waste disposal bags/bin liners to the lab/ward?

a11. Is there infectious waste treatment procedure carried out for such waste before disposal?

a12. Is there a (assigned) person/staff responsible to ensure waste is segregated and/or treated before disposed (waste and disposal system)?

a13. Is there regular training provided to waste handlers and assigned person/staff responsible for waste disposal system?

How often is this training?

a14. Is there some form of supervision on waste disposal system from Ministry? From NGO?

**Any further comments on availability and quality of diagnostic equipment.**

Data collected by:

Date:

1. For RDTs and items of essential infrastructure and equipment, facility staff were asked to self-report availability, which was then verified by observation. In the case of RDTs, a picture was taken of each device. In the case that tests were reported to be available but were found not to be usable, the device was reported as unavailable with a note made of why. For manual assays, such as malaria microscopy, which require a combination of equipment (microscope), reagents (giemsa stain), and consumables (for example microscopy slides) availability was based on reported availability by a designated person in charge of the laboratory. We then verified the available equipment, reagents and consumables for the most commonly reported available manual assays by observation. [↑](#endnote-ref-1)
2. Tests included in EDL but not included in Basic Package include: Albumin RDT; Bilirubin RDT; Hepatitis B e antigen RDT (HBeAG); HIV-related p24 antibody RDT; virological nucleaic acid POC; Cryptococcal antigen RDT test; Influenza A and B antigen detection RDT; Influenza A and B point of care nucleic acid test; Tuberculin skin (Mantoux) intradermal test. [↑](#endnote-ref-2)
3. Supply source was categorised as either “government” for items delivered to the District Health Management Team (DHMT), the local government body responsible for the organisation of primary care, (delivered to the DHMT either from the Central Medical Store or from national-level vertical disease programmes funded by the Global Fund to Fight AIDS, Tuberculosis and Malaria (from here on referred to as the Global Fund)), or “private” for supply sources that did not go through the DHMT, such as local pharmacies, private links to suppliers, or private organisations, such as religious NGOs. In some CHCs, microscopy slides were provided by the government, and reagents were supplied by private sources, in which case they were coded as a mixture of sources. [↑](#endnote-ref-3)
4. Additional items included in the WHO laboratory standards manual but for which no proxy was assessed in our survey included: ventilation; locked storage and secured areas; container for solvents and radiological waste; quality assurance systems. [↑](#endnote-ref-4)
5. Questions i-iv were added at later stage, gave these numbers so as not to confuse earlier numbering. [↑](#footnote-ref-1)
