# Supplementary material for: The “ready-to-hand” test: Diagnostic availability and usability in primary health care settings in Sierra Leone
Source: PLOS Glob Public Health. 2023 Feb 10;3(2):e0000604. doi: 10.1371/journal.pgph.0000604 (PMC10021322; doi:10.1371/journal.pgph.0000604)
Supplement: Attachment — Submitted filename: Response to the reviewers.docx [file pgph.0000604.s004.docx]

Dear Editors and Reviewers,

Many thanks for these helpful comments on our manuscript and suggestions for improvement. In particular, we found the suggestions for revisions to the introduction and restructuring of the results and discussion sections very useful when revising the manuscript and we hope that you find this version to be much improved.

We have provided a point-by-point response to each comments made by the reviewers below.

**Reviewer #1:**

This is a very comprehensive assessment of the availability of RDTs, laboratory infrastructure, and laboratory personnel at CHC in Sierra Leone. Well done.

Was the RDT and other equipment inventory physically viewed or counted during the interviews or based only upon verbal / written responses from the interviewee? Please add additional clarification in the Methods section.

*Thank you for these useful comments. We physically viewed the RDTs and other equipment during the visits to the CHCs. We added this to the Methods section – survey tools and variables.*

Also, minor edit in Table 2 - "Lightning" should be "Lighting"?

*Done.*

**Reviewer #2:**

One of the criteria for publication is that ‘PLOS Global Public Health does not copyedit accepted manuscripts, so the language in submitted articles must be clear, correct, and unambiguous’. It took me some time to click the YES button as part of my review. I do NOT mean to say the writers do not use English grammar properly, I DO mean to say that I think the paper can be significantly improved for the reader by doing a rewrite focused on telling a larger story. Currently the paper is too long (especially the introduction) and is a drip drip drip litany of all the things that are problematic with this or that rural or urban lab. The reader leaves with the sense that things are not well in Sierra Leone, but not with the larger story e.g. The presence of a trained laboratory scientist improves lab quality OR Rural labs in this province of Western Sierra Leone have a higher dependence on the private sector.

*Thank you for the useful feedback. We have adjusted several sections (as also indicated in our answers to the more detailed feedback below) to make the introduction more succinct and to highlight the key takeaway points from each section. In the discussion section we have emphasised the encouraging finding that laboratory scientists are going the extra mile to make diagnostics available.* *We have also added key recommendations for policy and resource-investment in Sierra Leone in the conclusion.*

Detailed feedback.
1. Title – The meaning of the phrase ‘Ready to Hand’ is not obvious until it is defined also it does not communicate across different types of English speakers. Consider using just “Diagnostic availability and usability in primary health care settings in Sierra Leone” as the title.

*We believe it is important to include the notion of ‘ready to hand’ in the title because it provides a social science lens to assess what availability means, beyond the physical presence of a test. We believe this captures the main contribution of the article. We have explained what the notion of ‘ready to hand’ means in the abstract, and have amended the introduction so that it is discussed earlier in the text. However, we are happy to defer to the Journal Editors on this point if they feel the current title does not fit the conventions of the journal.*

Reviewer #2
1. Introduction
a. Too long
b. The first two paragraphs do not actually introcude the relevant subject to the reader. Rather it is a trope of dependency and ‘lack’ that many of us in the Global Health world – including me- have come to depend on. Resist the temptation. Delete these two paragraphs. It will bring the reader into the relevant material faster. Start with ‘Since the publication of the EDL…

*We agree entirely with this concern. However, we believe that providing some contextual historical background in relation to the omission of diagnostics from global health policy is necessary in order to explain why this study is important. We do not want to assume that readers will already have this background knowledge. However, taking the reviewer’s suggestion on board, we have reduced the first two paragraphs to one paragraph to make it more succinct and to introduce sooner recent work to improve diagnostic availability in LMICs, including the introduction of the EDL.*

2. Methods – Good

3. Results
a. Litany of problems, larger messages / themes were not clear. Yes I know this is the result section, not the discussion but if there is a larger thematic approach it will affect what gets highlighted.

*We have included an introductory paragraph in the results to highlight the structuring of the findings in line with our focus on the “ready to hand” test.*

b. In the figures, consider using other none-colorimetric ways of differentiating your various groups. Ways such as cross-hatching or dots. The advantage of this is that they still communicate even if one doesn’t have a color printer.

*We have considered both suggestions, but when adapting the figures to include shades of black and white, or dots/cross-hatching the figures becomes less clear. In the trade-off between visual accessibility on screen Vs in print, we elected to choose screen accessibility and have left the figures how they are. However, we are happy to defer to the editor’s opinion on this.*

c. The percentage with tap water was 59%. Why was the denominator 39 instead of 40?

*Thank you for spotting this omission, we have adjusted the denominator to 40. We also found several errors in the data included in the tables, which had not been updated to reflect our cleaned data-set. We have now corrected these.*

d. Consider expanding the paragraph that starts ‘ There was a greater dependency… ‘. Also consider putting it in the Discussion. I think this finding is important and will be of wide interest to your Global Health readership

*We moved this paragraph to the discussion on page 28, where we provide additional information about the differences in supply systems within the districts.*

4. Discussion

a. One gap was ..when items are sourced privately, how do the lab staff recoup their money? Same question for when they pay for transporting specimens. Are the costs being transferred to the patients?

*In those situations, patients are being charged to recoup the costs. We have clarified this on page 27.*
