# Supplementary material for: The “ready-to-hand” test: Diagnostic availability and usability in primary health care settings in Sierra Leone
Source: PLOS Glob Public Health. 2023 Feb 10;3(2):e0000604. doi: 10.1371/journal.pgph.0000604 (PMC10021322; doi:10.1371/journal.pgph.0000604)
Supplement: Attachment — Submitted filename: Response to reviewers.docx [file pgph.0000604.s005.docx]

08.12.22

Dear Editor(s),

Thank you for the opportunity to resubmit our manuscript. We have addressed all remaining comments and included our responses to the questions raised below.

We look forward to hearing from you.

Alice Street

p. 7: “The Basic Package included 27 tests for the CHC level, 17 of which were RDTs in the form of lateral flow dipsticks, cassettes or handheld devices”. **However, Figure 1 only lists 16 RDTs? If we include the add-on cholera RDT, should Figure 1 list 18 RDTs?**

**This is our mistake. The basic package listed 16 RDTs. With the cholera test, that makes 17 RDTs. We have corrected the text accordingly.**

p. 9: “Fisher’s exact test was used if expected frequencies had a value of >5”. **Should the value be <5 (instead of >5)?**

**Yes, the value should be <5. We have corrected the text accordingly.**

p. 21: “70% of CHCs (n/N = 28/40) had electricity in all parts of the facility but less than half of the CHCs in both districts had a working standby generator available 40.0%, n/N = 6/25 in rural district, 44.0%, n/N = 11/25 in urban district). **Missing bracket before 40%.**

**Done**

p. 24: “One RDT type, for Hepatitis B, was dependent entirely on private supply in both districts where is was available” **correct typo**

**Done (changed ‘is’ to ‘it’)**

p. 25: “Three manual assay tests (TB Sputum Microscopy, Blood Microscopy, Fungal Identification) were exclusively supplied by government sources. Likewise, three others (Widal Test, Blood Typing, White Blood Cell Count) were exclusively supplied by private sources”. **Suggest replacing “likewise” with a “while” construction as there is no conceptual similarity other than the same number.**

**Done, we replaced ‘likewise’ for ‘while’.**

p. 29: “The greater dependence on private supply chains in the in Rural district was possibly related to…” **correct typo**

**Done.**

p. 32: “and that it is important to focus instead on whether particular particular tests are “doable” **correct typo**

**Done, deleted the second particular.**

p. 33: “We did not include quality assurance systems in our survey because no external quality assessments systems were running in the districts that we surveyed at the time of data collection (quality assurance systems were provided at secondary level laboratories, and country-wide audits of laboratory quality were undertaken” **missing bracket**

**Done.**

**General comments:**

- All figures can be in RGB as long as the B&W printout is clear.

**We can confirm the B&W printout is clear and so will use RGB**

- There are many sentences starting with a percentage – try to reword or, if unwieldy, spell out the number.

**Thank you for this helpful comment, we made several edits on pages 17, 21, 22.**

- Spell out each acronym first time of use – e.g. CDC, MOHS, EVD, DHMT

**Done, we have spelled out each acronym at first time is use.**

- Please review your reference list to ensure that it is complete and correct. If you have cited papers that have been retracted, please include the rationale for doing so in the manuscript text, or remove these references and replace them with relevant current references. Any changes to the reference list should be mentioned in the rebuttal letter that accompanies your revised manuscript. If you need to cite a retracted article, indicate the article’s retracted status in the References list and also include a citation and full reference for the retraction notice.

**During our previous revision we were asked by the reviewer and editor to make our manuscript more succinct, hence we cut several sections in the introduction, which is why some references were cut in the previous revision. No papers included in previous versions of the paper have since been retracted. We have made several corrections to the citations included in the paper to ensure they comply with the style guidelines and have de-anonymised those references by authors of this paper and which were previously anonymised.**
